# Review of Journal of Cardiovascular Magnetic Resonance (JCMR) 2015-2016 and transition of the JCMR office to Boston

**DOI:** 10.1186/s12968-017-0423-x

**Published:** 2017-12-28

**Authors:** Warren J. Manning

**Affiliations:** From the Journal of Cardiovascular Magnetic Resonance Editorial Office and the Beth Israel Deaconess Medical Center, Harvard Medical School, Boston, MA USA

## Abstract

The *Journal of Cardiovascular Magnetic Resonance (JCMR)* is the official publication of the Society for Cardiovascular Magnetic Resonance (SCMR). In 2016, the *JCMR published* 93 manuscripts, including 80 research papers, 6 reviews, 5 technical notes, 1 protocol, and 1 case report. The number of manuscripts published was similar to 2015 though with a 12% increase in manuscript submissions to an all-time high of 369. This reflects a decrease in the overall acceptance rate to <25% (excluding solicited reviews). The quality of submissions to JCMR continues to be high. The 2016 *JCMR* Impact Factor (which is published in June 2016 by Thomson Reuters) was steady at 5.601 (vs. 5.71 for 2015; as published in June 2016), which is the second highest impact factor ever recorded for *JCMR*. The 2016 impact factor means that the *JCMR* papers that were published in 2014 and 2015 were on-average cited 5.71 times in 2016.

In accordance with Open-Access publishing of Biomed Central, the *JCMR* articles are published on-line in the order that they are accepted with no collating of the articles into sections or special thematic issues. For this reason, over the years, the Editors have felt that it is useful to annually summarize the publications into broad areas of interest or themes, so that readers can view areas of interest in a single article in relation to each other and other recent *JCMR* articles. The papers are presented in broad themes with previously published *JCMR* papers to guide continuity of thought in the journal. In addition, I have elected to open this publication with information for the readership regarding the transition of the *JCMR* editorial office to the Beth Israel Deaconess Medical Center, Boston and the editorial process.

Though there is an author publication charge (APC) associated with open-access to cover the publisher’s expenses, this format provides a much wider distribution/availability of the author’s work and greater manuscript citation. For SCMR members, there is a substantial discount in the APC. I hope that you will continue to send your high quality manuscripts to *JCMR* for consideration. Importantly, I also ask that you consider referencing recent *JCMR* publications in your submissions to the *JCMR* and elsewhere as these contribute to our impact factor. I also thank our dedicated Associate Editors, Guest Editors, and reviewers for their many efforts to ensure that the review process occurs in a timely and responsible manner and that the *JCMR* continues to be recognized as the leading publication in our field.

## *JCMR* transition to Boston/editorial process

At the end of December 2016, the editorial office of the Journal of Cardiovascular Magnetic Resonance (*JCMR*) moved to the Beth Israel Deaconess Medical Center, Boston under the leadership of Dr. Warren J. Manning – former Deputy Editor of the *JCMR* and current editor-in-chief. Manuscripts that were in processing at the Royal Brompton Hospital *JCMR* office continued to be handled by that office and all *JCMR* activities under Dr. Pennell’s leadership and editorial team ceased at the end of April 2017.

### JCMR leadership/communication

**Fig. 1:**
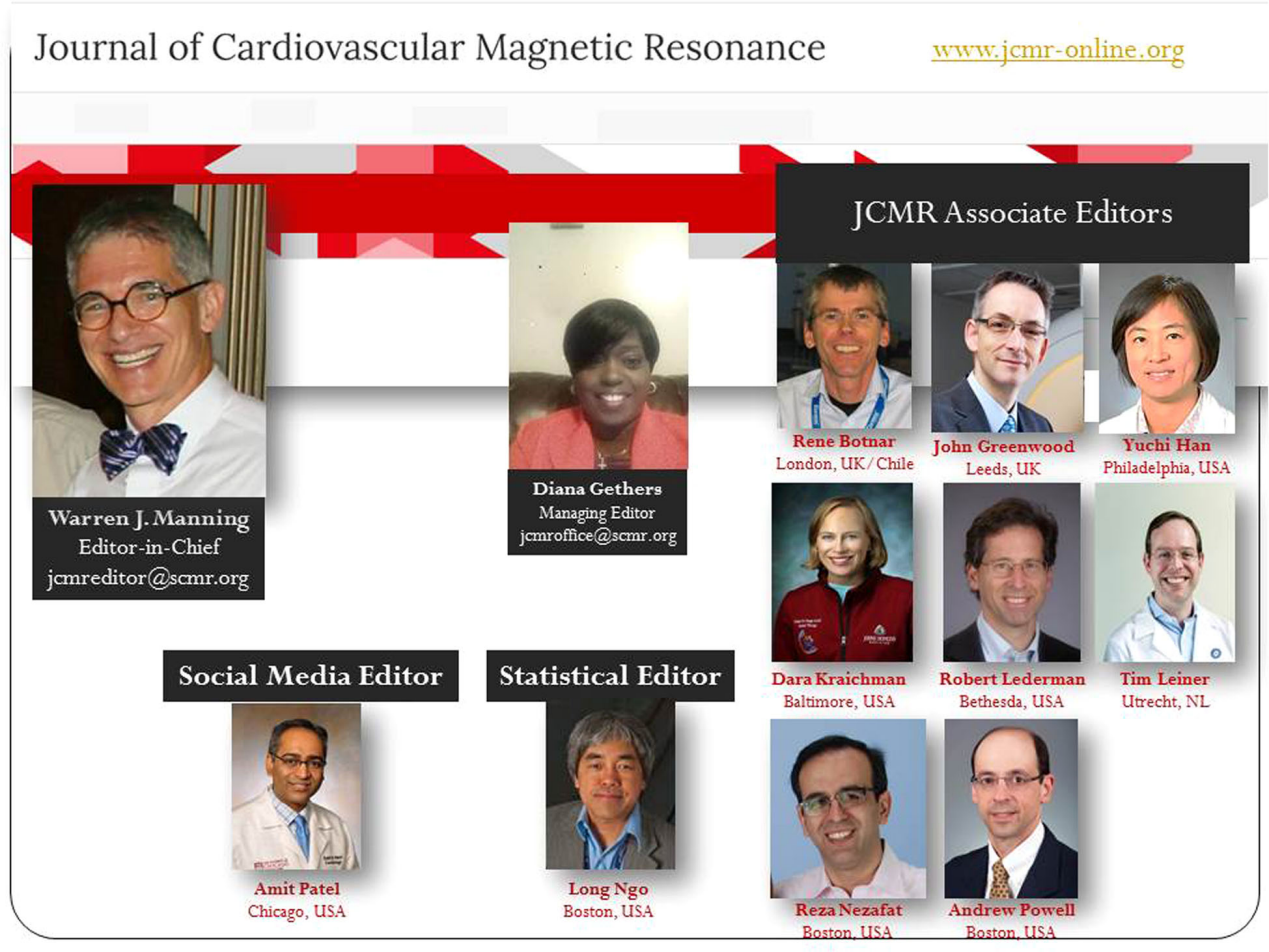
2017 *JCMR* Editorial team

As compared with the prior two editor’s handling of all editor/associate editor activities at their “home” institution, the current *JCMR* Associate Editors are diverse in geography and include (Fig. 1) Drs. Rene Botnar (Kings College, London, UK/Chile), John Greenwood (University of Leeds, UK), Yuchi Han (University of Pennsylvania Health System, USA), Dara Kraitchman (Johns Hopkins University School of Medicine, USA), Robert Lederman (National Heart, Lung, and Blood Institute, USA), Tim Leiner (UMC Utrecht, The Netherlands), Reza Nezafat (Beth Israel Deaconess Medical Center/Harvard Medical School, USA) and Andrew Powell (Children’s Hospital Boston/Harvard Medical School, USA). In addition, Dr. Long Ngo (Beth Israel Deaconess Medical Center/Harvard Medical School, USA) serves as the *JCMR* statistical editor and Dr. Amit Patel (University of Chicago, USA) serves as our social media editor. Ms. Gifty Addae served as our managing editor until August 2017, after which Mrs. Diana Gethers took on the responsibilities as our managing editor. Despite the change in managing editor leadership, the *JCMR* office email address continues to be jcmroffice@scmr.org.

To facilitate communication in an era of constant change, a generic email address between the *JCMR* and readership was instituted including the use of** jcmreditor@scmr.org **for the editor-in-chief and **jcmroffice@scmr.org **for the managing editor.

## *JCMR* publication/review process

The *JCMR* is the official publication of the Society for Cardiovascular Magnetic Resonance (SCMR) and has been published in open access format for a decade through BioMed Central (BMC) as the publisher. All manuscripts are submitted through the www.jcmr-online.biomedcentral.com website.

After initial confirmation that the manuscript is in the appropriate format (abstract, text, references, figures, tables, etc.) by the BMC office, the manuscript is sent for initial review to the Boston editorial office. Within 48 h, the manuscript is assessed for its appropriateness for the *JCMR* readership and a determination as to its overall likely priority for publication. Approximately 20% of submitted manuscripts are returned to the author at this stage and without review so as to expedite their submission to another more appropriate journal. Authors are often offered the opportunity to submit their work to another BMC open access publication. Nearly 50% of authors take advantage of this option.

Reviewer assignments are then requested for manuscripts that are deemed worthy of further review and the manuscript is assigned to one of our Associate Editors. Reviews are requested within 2 weeks and reviewers are asked to follow specific submission guidelines as outlined in their offer letter. We are fortunate to have over 1162 registered reviewers, but are continually looking to expand our reviewer pool, engaging younger members/innovators of the CMR field. If you are interested in becoming a *JCMR* reviewer, please sign up to be a reviewer at **https://www.surveymonkey.com/r/JCMRreview** or contact our managing editor, Diana Gethers [jcmroffice@scmr.org].

When at least two reviews have been received, the Associate Editor presents the manuscript at our Web-Ex Associate Editorial board meeting held every Tuesday morning from 9:30-10:30 am EST. [When I am out of town/away, the Associate Editors continue to meet at that time so as to not delay the publication process.] At each meeting, 4-12 manuscripts may be discussed. Decisions include

1. Accept
2. Minor revision – no new experiments are needed, relatively minor text changes or analysis requested, 30 day turn-around; >98% acceptance is anticipated
3. Major revision – substantial text and or analysis needed, a few experiments; 90 day turn-around; ~60% acceptance is anticipated
4. De novo resubmission – substantial new experiments/analysis are needed; unlimited turn-around; ~35% acceptance is anticipated
5. Decline/reject

Our target goal is than 60% of manuscripts will have a submission to *first* decision within 31 days, a process that is very dependent on our receipt of timely reviews. If the two reviews markedly differ in their assessment (~25% of the time), a third reviewer is often solicited – a process than can add up to a month to the overall review process. In rare instances, a fourth review may be requested. When added reviews are requested, we try to alert the corresponding author that there will be a delay. Revisions may (major some minor) or may not (most minor) be sent to the prior or new reviewers. Denovo resubmissions are usually sent to the original manuscript reviewers.

### Conflicts of interest

Rather than having a single deputy editor to handle “conflict of interest “manuscripts submitted by members of the Associate Editorial team (or their close associates), these manuscripts are handled by a Guest Editor. The activities of the Guest Editor are conducted outside/independent of the Boston editorial office. If accepted, the Guest Editor is disclosed in the publication.

### Social media

Initiated by our formed editor-in-chief, Professor Dudley Pennell, the *JCMR* is active on Twitter with the handle “**JournalofCMR**”. This relationship is coordinated by Dr. Amit Patel. As of mid-November 2016, our Twitter statistics indicate that we have 990 followers and have had 153,070 total impressions; 6848 engagements, 723 retweets, 1629 URL clicks, and 2377 media views. In 2017, our engagement rate has increased from 1.30 to 1.51.

### Continuing medical education

New for 2017 is our development of a process for our readership to receive Continuing Medical Education (CME) credit for *JCMR* publications through the SCMR. Ongoing level II/III certification in CMR requires CME credit. The first CME offering was for a manuscript by Chan and colleagues on the tissue characterization of cardiac neoplasm and thrombus [1]. We had 2 offerings in 2017 and hope to have quarterly offerings in 2018. In 2018, we also hope to offer CME credit for our reviewers. Stay tuned!

### Gerald M. Pohost award/Dudley Pennell award

**Fig. 2:**
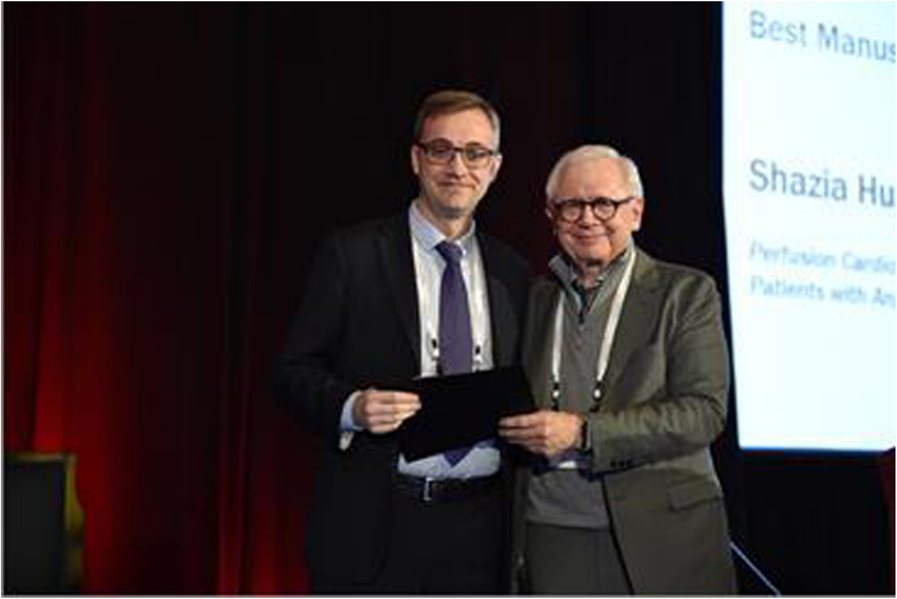
The 2017 Gerald M. Pohost Award is presented at the 2017 annual meeting of the SCMR

For the past decade, the SCMR has presented the Gerald M. Pohost Award to that manuscript identified by the *JCMR* editorial board to be *the most important JCMR publication* of the prior year. Dr. Pohost was the first *JCMR* editor-in-chief. The 2017 Gerald M. Pohost Award was presented to Dr. Shazia Hussain and colleagues for their publication “Perfusion cardiovascular magnetic resonance and fractional flow reserve in patients with angiographic multi-vessel coronary artery disease.” [2][Fig. 2]. Beginning with the 2018 SCMR Annual Meeting, in recognition to our 2nd editor-in-chief and the tremendous rise in the *JCMR* Impact Factor during his tenure, we will also be presenting the inaugural Dudley Pennell Award to that original research manuscript which has received the highest number of citations (thereby contributing to the *JCMR* impact factor) for the calendar year 3 years prior to the award (i.e, 2018 Dudley Pennell Prize will be awarded to that original research manuscript published in calendar year 2014).

**2016 Publications:**

Listed below are summaries of the 2015/2016 *JCMR* publications presented in thematic format with the reference (2015 publications) [3] and brief manuscript synopsis (2016 publications).

## Cardiomyopathies

Cardiac phenotyping continues to be a primary clinical indication for CMR, and has become mainstream in the common cardiomyopathies including hypertrophic cardiomyopathy (HCM; [4, 5]), Anderson-Fabry Disease [6, 7], dilated cardiomyopathy [8–11], left ventricular non-compaction (LVNC [12], myocarditis [13–16], Chagas disease [17], cardiac siderosis [18] and right ventricular dysfunction due to pulmonary hypertension [19, 20].

### Hypertrophic cardiomyopathy

**Microvascular ischemia in hypertrophic cardiomyopathy: new insights from high-resolution combined quantification of perfusion and late gadolinium enhancement.**

Microvascular ischemia is one of the hallmarks of hypertrophic cardiomyopathy (HCM) and has been associated with poor outcomes. Villa and co-workers [21] examined 30 HCM patients with non-obstructive epicardial coronary arteries with Fermi constrained quantitative perfusion analysis on segmental and high-resolution data. High resolution quantification proved to be more sensitive for the detection of microvascular ischemia in comparison with segmental analysis. Areas of late gadolinium enhancement (LGE) were associated with a significant reduction in myocardial perfusion reserve (MPR) leading to an *overestimation* of the total ischemic burden. Using an MPR of 1.5, the presence of LGE was found to lead to an ischemic burden overestimation of 28%.

**Cardiovascular magnetic resonance of mitral valve length in hypertrophic cardiomyopathy.**

Prior data suggest that mitral leaflet elongation in hypertrophic cardiomyopathy (HCM). Tarkiainen et al. [22] studied 47 HCM subjects with Q1061X mutation in the gene encoding MYBPC3 and 20 relatives without the mutation. Subjects with the MYBPC3-Q1061X mutation included 32 with left ventricular (LV) hypertrophy. The absolute posterior mitral leaflets was longer in the MYBPC3-Q1061X patients, but was not significant when the posterior leaflet length was indexed for body surface area. There was no difference in posterior mitral leaflet length in mutations carriers without LV hypertrophy and healthy controls.

### Anderson-Fabry disease

**Cardiovascular magnetic resonance demonstration of the spectrum of morphological phenotypes and patterns of myocardial scarring in Anderson-Fabry disease.**

Anderson-Fabry disease can mimic hypertrophic cardiomyopathy (HCM) on transthoracic echocardiography. Deva and co-workers [23] studied 39 Anderson-Fabry patients (20 M) and found that 44% had concentric wall thickening. In addition, 44% had pathologic scar/late gadolinium enhancement (LGE) of which 76% had typical inferolateral midwall LGE. Patients with elevated left ventricular (LV) mass index had more ventricular arrhythmia and sustained ventricular tachycardia.

### Amyloid Cardiomyopathy

**Regional myocardial microvascular dysfunction in cardiac amyloid light-chain amyloidosis: assessment with 3T cardiovascular magnetic resonance.**

Coronary microvascular dysfunction is highly prevalent in patients with cardiac light-chain (AL) amyloidosis. In this study by Li and co-workers [24] performed regional myocardial perfusion CMR in 32 patients with biopsy proven cardiac AL amyloidosis and systolic dysfunction, 21 patients with cardiac AL amyloidosis and preserved systolic function, and 25 healthy subjects. Patients with cardiac AL amyloidosis had significantly reduced first pass perfusion upslope and maximal signal intensity. Compared with cardiac AL amyloid patients with preserved systolic function, those with depressed systolic function has a longer time to maximum signal intensity in the basal and mid-ventricular segments and lower upslope in the basal, mid, and apical segments.

### Non-ischemic cardiomyopathies:

**Mechanical effects of left ventricular midwall fibrosis in non-ischemic cardiomyopathy.**

Left ventricular mid-wall fibrosis occurs in a quarter of patients with a non-ischemic cardiomyopathy and is associated with high risk of pump failure. Taylor and colleagues [25] examined 116 patients who underwent late gadolinium enhancement (LGE) CMR with feature tracking CMR to assess myocardial deformation. Despite similar left ventricular ejection fraction (LVEF), patients with mid-wall fibrosis had lower global circumferential strain, strain rate, and torsion, but similar longitudinal and radial strain.

**Analyzing myocardial torsion based on tissue phase mapping cardiovascular magnetic resonance.**

Chitiboi et al. [26] performed tissue phase mapping (base, mid, apical short axis) and late gadolinium enhancement (LGE) in 27 patients with non-ischemic cardiomyopathy and tissue phase mapping in 14 healthy subjects. Maximal myocardial torsion was significantly lower for the cardiomyopathic patients. Global myocardial torsion showed a positive correlation with left ventricular (LV) ejection fraction (LVEF). Moreover, endocardial torsion was significantly higher than epicardial torsion for normal LVEF subjects.

**Relationship between native papillary muscle T1 time and severity of functional mitral regurgitation in patients with non-ischemic dilated cardiomyopathy.**

Functional mitral regurgitation is one of the severe complications of non-ischemic dilated cardiomyopathy. Kato and co-workers [27] studied 40 patients with non-ischemic cardiomyopathy and 20 healthy controls and found both papillary muscle native T1 were significantly elevated in the cardiomyopathy group with mitral regurgitation v. those without mitral regurgitation. Multivariate linear regression analysis showed that papillary muscle native T1 correlated with mitral regurgitant fraction. Elevated papillary muscle native T1 was also associated with larger diameter, longer length, and decreased papillary muscle shortening.

**Objective criteria for septal fibrosis in non-ischemic dilated cardiomyopathy: validation for the prediction of future cardiovascular events.**

Expert subjective mid-wall septal fibrosis on late gadolinium enhancement (LGE) CM R has been shown to predict major adverse cardiovascular outcomes. Mikami et al. [28] sought to establish objective criteria for non-experts and studied 118 consecutive patients with a non-ischemic cardiomyopathy and septal fibrosis identified by an expert reader. CMR naïve readers performed signal threshold-based LGE quantification. At nearly 2 year follow-up, 17% of patients had a primary composite outcome. Expert visual scoring identified 55 patients with septal fibrosis. Receiver operator curve analysis demonstrated the optimal threshold for prediction of primary outcome were 5SD; a level that also was the strongest independent predictor of the primary outcome and provided improved risk reclassification beyond left ventricular ejection fraction (LVEF).

### Muscular dystrophies

**Increased myocardial native T1 and extracellular volume in patients with Duchenne muscular dystrophy.**

Duchenne’s muscular dystrophy cardiomyopathy is a progressive incurable disorder. Detection of subclinical disease may be useful for early intervention and for monitoring disease specific therapies. In this prospective study, Soslow and colleagues [29] examined native T1 and extracellular volume fraction (ECV) in 31 Duchenne’s patients and 11 healthy controls. They found that even in the Duchenne’s subset with a normal left ventricular ejection fraction (LVEF) and no late gadolinium enhancement (LGE), Duchenne participants had a greater native T1 and greater ECV.

**Identification of cardiomyopathy associated circulating miRNA biomarkers in patients with muscular dystrophy using a complementary cardiovascular magnetic resonance and plasma profiling approach.**

Duchenne and Becker muscular dystrophy are x-linked recessive neuromuscular disorders. In this study, Becker et al. [30] prospectively examined 63 male patients with known muscular dystrophy and 26 age-matched healthy controls. Impaired left ventricular (LV) systolic function (ejection fraction <55%) and a non-ischemic late gadolinium enhancement (LGE) pattern were found in 46% and 76% of muscular dystrophy patients, respectively. A significant upregulation of circulating miRNAs miR-222, miR-26a, and miR-378-5p were independent predictors for LGE.

**Myocardial late gadolinium enhancement is associated with clinical presentation in Duchenne muscular dystrophy.**

Duchenne’s muscular dystrophy is an x-linked recessive disease that occurs in males leading to immobility and death in early adulthood. Female carriers are generally asymptomatic, but sometimes develop a dilated cardiomyopathy. Wexberg and colleagues [31] studied 20 Duchenne’s carriers, the majority of which (90%) were asymptomatic, yet nearly half had late gadolinium enhancement (LGE), all of whom were NYHA class II or III. LGE positive carriers had lower left ventricular (LV) ejection fraction, higher creatine phosphokinase and shorter 6-min walk test.

**Native T1 values identify myocardial changes and disease severity in patients with Duchenne muscular dystrophy.**

Duchenne’s muscular dystrophy is an x-linked, inherited disorder that leads to a dilated cardiomyopathy with variable onset and progression. Olivieri and co-workers [32] examined 20 boys with Duchenne’s and 16 age and gender matched controls and found 60% of the Duchenne subjects and late gadolinium enhancement (LGE) of the lateral wall. Native T1 and extracellular volume fraction (ECV) were also significantly higher in the Duchenne cohort with both MOLLI and SASHA T1 sequences. ECV demonstrated the ability to predict the presence of LGE, but could not distinguish between healthy controls and Duchenne’s without LGE.

### Left ventricular non-compaction

**Quantification of left ventricular trabeculae using cardiovascular magnetic resonance for the diagnosis of left ventricular non-compaction: evaluation of trabecular volume and refined semi-quantitative criteria.**

There is no consensus for the diagnosis of left ventricular non-compaction (LVNC). In this study Choi and co-workers [33] retrospectively identified 145 subjects with mild to severe left ventricular (LV) trabeculations, 24 patients with isolated LVNC, 33 patients with non-isolated LVNC, and 30 patients with dilated cardiomyopathy. The patients with isolated LVNC had trabecular LV volume that was 1.4× greater than those with a dilated cardiomyopathy and 1.7× greater than the healthy controls. There was no difference in the percent trabecular volume between the isolated LVNC and dilated cardiomyopathy groups. A percent trabecular volume threshold of >34.6% had a specificity of 89.7% and sensitivity of 66.1% for LVNC.

### Chagas disease

**Chagas’ heart disease: gender differences in myocardial damage assessed by cardiovascular magnetic resonance.**

A male-related higher incidence of cardiovascular morbidity and mortality has been described with Chagas heart disease. Assunacao and colleagues [34] retrospectively identified 62 seropositive Chagas’ heart disease patients and low probability of coronary artery disease (CAD) referred for CMR at 1.5T. There was a strong correlation between left ventricular (LV) ejection fraction (LVEF) and late gadolinium enhancement (LGE) with men showing greater LGE (including transmural pattern) and lower LVEF. After adjustment for confounders, male gender remained associated with LV dysfunction with 5% of the effect mediated by LGE.

#### Pompe disease

**Pattern and prognostic value of cardiac involvement in patients with late-onset Pompe Disease: a comprehensive cardiovascular magnetic resonance approach.**

Pompe disease is an autosomal recessive disorder caused by a deficiency of the lysosomal alpha-1,4-glucosidease which leads to an accumulation of glycogen in target tissue and progressive organ failure. Boentert and co-workers [35] studied 17 patients with genetically proven late-onset Pompe disease (50 +/− 18 years; 11 men) and 18 age and gender matched controls with CMR. They found that all Pompe Disease patients had normal left ventricular (LV) and right ventricular (RV) volumes and ejection fraction and feature tracking measures of left ventricular strain. Three (18%) of the Pompe Disease patients had a non-ischemic late gadolinium enhancement (LGE) pattern and 21% had an elevated extracellular volume fraction (ECV).

### Obesity

**Cardiac remodeling and dysfunction in childhood obesity: a cardiovascular magnetic resonance study.**

Obesity affects nearly 20% of children and is associated with an increased premature death. In this study, Jing and colleagues [36] performed CMR in 41 obese/overweight and 29 healthy weight children. After adjusting for age, LV mass index was 23% greater and the myocardial wall was 10-% thicker in the obese/overweight cohort. Nearly a quarter of the obese/overweight cohort had concentric hypertrophy. Peak longitudinal strain and circumferential strain showed a significant relationship with the type of remodeling and were most impaired in children with concentric hypertrophy.

### Connective tissue disorders

**Evaluation of myocardial involvement in patients with connective tissue disorders: a multi-parametric cardiovascular magnetic resonance study.**

Arrhythmias and heart failure are sometimes found in patients with connective tissue disorders and myocardial involvement. Mayr and co-workers [37] prospectively performed CMR in 40 patients with connective tissue disorder and 20 healthy controls. They found that 18% of connective tissue disorder sometimes displayed late gadolinium enhancement (LGE). The connective tissue disorder group also had a greater extracellular volume (ECV), T2, and native T1 with lower post-contrast T1.

**Cardiovascular magnetic resonance patterns of biopsy proven cardiac involvement in systemic sclerosis.**

Systemic sclerosis may sometimes involve the myocardium. In this retrospective study, Krumm et al. [38] identified 20 patients with biopsy proved cardiac involvement in systemic sclerosis and found 45% had a moderate (>5 mm) or greater pericardial effusion; 95% had reduced left ventricular (LV) or right ventricular (RV) systolic function and 70% of patients had late gadolinium enhancement (LGE). Seven (35%) of patients had all three categories of abnormalities.

### Arrhythmogenic right ventricular cardiomyopathy

**Ventricular structure in ARVC: going beyond volumes as a measure of risk.**

Altered right ventricular (RV) anatomy and function is an important feature of the arrhythmogenic right ventricular cardiomyopathy (ARVC) phenotype. In this retrospective, cross-sectional study, McLeod and co-workers [39] examined CMR short axis cines in 27 ARVC patients and 21 asymptomatic control subjects. They found that the ARVC cohort was more likely to have global dilation/shrinking of both ventricles (44%), elongation/shortening of the RV (15%), tilting of the septum (10%), shortening/lengthening of both ventricles (7%), and bulging/shortening of both the RV inflow and outflow (5%). Classification of ARVC v. controls using shape modes had a sensitivity and specificity of 96% and 81%, respectively.

### Syndrome X

**Coronary microvascular function and myocardial fibrosis in women with angina pectoris and no obstructive coronary artery disease: the iPOWER study.**

Despite the absence of obstructive coronary artery disease, women with angina pectoris have a poor prognosis due to microvascular disease. Mygind et al. [40] performed CMR and coronary flow velocity reserve in 64 women with angina a no obstructive coronary artery disease. None had focal late gadolinium enhancement. No significant correlations were found between coronary flow velocity reserve and extracellular volume fraction (ECV) or native T1. In addition, no relationship was seen between myocardial blood flow reserve and ECV or native T1.

**Cost Effectiveness/Databases/Normative Values.**

With the increasing focus on the growing societal burden of health care costs, it is imperative that CMR demonstrate safety [41], value/cost effectiveness/appropriateness [42, 43]. Increasing large databases to define normative CMR parameters [44–49] are also reported.

### Cost effectiveness

**Cost-minimization analysis of three decision strategies for cardiac revascularization: results of the “suspected CAD” cohort of the European Cardiovascular Magnetic Resonance Registry.**

Coronary artery disease (CAD) continues to be the leading cause of morbidity and mortality in the developed world. While perfusion CMR is generally accepted as clinically useful, cost-effectiveness data are scarce. Moschetti et al. [50] examined 3647 patients with suspected CAD from the European Cardiovascular Magnetic Resonance Registry and examined costs over a 1 year period for CMR, x-ray angiography with/without fractional flow reserve, revascularization, and complications. Coronary revascularization was performed in 6.2% of the entire group, including 4.5% and 12.9% of those with atypical and typical chest pain, respectively. A CMR + xray angiography strategy reduced costs by 14-34% vs. x-ray angiography + fractional flow reserve strategy and by >50% when compared with x-ray angiography only strategy.

### CMR in guidelines

**Role of cardiovascular magnetic resonance in the guidelines of the European Society of Cardiology.**

Despite enthusiasm for CMR, its application in Europe is quite diverse. Von Knobelsdorff-Brenkenhoff and Schulz-Menger [51] examined 29 European Society of Cardiology guidelines for their recommendation for CMR and found that only 14/54% of the guidelines contained a specific recommendation regarding CMR. The 14 guidelines had 63 CMR recommendations including 39 class-I recommendations, 12 class II-a recommendations, 10 class-IIb recommendations, and 2 class III recommendations. Nearly 2/3rds of the recommendations were level of evidence C with only 10% level of evidence A. The most common guidelines were for stable coronary artery disease, aortic disease, and hypertrophic cardiomyopathy (HCM).

### UK biobank CMR substudy

**UK Biobank’s cardiovascular magnetic resonance protocol.**

The United Kingdom’s Biobank’s CMR substudy aims to perform CMR studies in 100,000 individuals from the entire 500,000 Biobank cohort of 40-69 year-olds. In this report, Petersen and co-colleagues [52] describe the CMR protocol applied in the UK Biobank’s pilot phase which will be extended to three centers. The CMR protocol includes white blood anatomy (sagittal, coronal and transverse), cine (long axis, ventricular short axis, coronal left ventricular outflow tract), strain/tagging, aortic valve flow and parametric native T1.

### Normative values - anatomy

**Pulmonary artery diameters, cross sectional areas and area changes measured by cine cardiovascular magnetic resonance in healthy volunteers.**

Normal diastolic and systolic cross-sectional area of the left main pulmonary artery and its branches are unknown. Burman and co-workers [53] recruited 120 healthy subjects, including 20 (10 males, 10 females) in each decile between 20 and 79 years. Cross sectional areas were rarely circular. Diastolic diameters increased with body surface area (BSA) and age and there was a decrease in distension with age. Normative plots for each decile and gender are provided.

**3D black blood VISTA vessel wall cardiovascular magnetic resonance of the thoracic aorta in young, healthy adults: reproducibility and implications for efficacy trial sample sizes: a cross-sectional study.**

Preclinical detection of atherosclerosis enables personalized preventive strategies and CMR is an attractive platform for such detection, but data on 3T black blood imaging is lacking. Eikendal and co-workers [54] performed 3T T1 weighted black blood VISTA vessel wall imaging of the aorta in 20 healthy young adults and found excellent inter-scan and inter-rater, and inter-rater data for aortic lumen, total vessel and wall area as well as mean and maximal wall thickness data with sample size estimates required to detect a 5% difference of 203, 126, 136, 68 and 153, respectively.

**Cardiovascular magnetic resonance reference ranges for the heart and aorta in Chinese at 3T.**

CMR reference ranges have not been well established in Chinese. Le and colleagues [55] performed 3T CMR n 180 healthy Singapore Chinese (20-69 years, 91 men) with image analysis by two observers. Left ventricular (LV) mass was similar in both systole and diastole and stroke volume was similar for the LV and right ventricle (RV). Indexed LV mass was greater in men while indexed atrial sizes and aortic root dimensions were similar. Overall, only 2% of total LV mass was comprised of papillary muscle mass.

**Age dependence of pulmonary artery blood flow measured by 4D flow cardiovascular magnetic resonance: results of a population-based study.**

Population based pulmonary artery blood flow is unknown. In this prospective study, Wehrum et al. [56] studied 126 age-stratified subjects (at least 20 subjects (10 M/10F) per decade) and found time-to-peak systolic antegrade flow was shorter and peak and average velocities and flow volumes were lower in older subjects. At the end of systole, retrograde flow in the main pulmonary artery was present in all but one subject.

**Relationship between body composition and left ventricular geometry using three dimensional cardiovascular magnetic resonance.**

Obesity is associated with alterations in left ventricular (LV) mass and volume. In this observational study, Corden and colleagues [57] studied 1530 subjects (mean age 41.3 years; 55% female) without known cardiovascular disease (CVD). LV mass was positively associated with fat mass in women but not in men. Male fat mass was strongly associated with concentric *increase* in relative wall thickness and *reduced* mid-ventricular cavity size.

**Between cardiac deformation parameters measured by cardiovascular magnetic resonance and aerobic fitness in endurance athletes.**

Athletic training leads to remodeling of both ventricles with increased myocardial mass and biventricular cavity dilation. In this study, Swoboda and co-workers [58] performed CMR tagging in 35 endurance athletes and 35 age and sex matched controls at 3T. LV circumferential strain, twist and torsion, late diastolic longitudinal strain rate and RV peak longitudinal strain, early and late diastolic longitudinal strain rate were all lower in athletes, but on multivariable linear regression, on LV torsion had a significant association with lactate threshold and only RV longitudinal late diastolic strain had a significant association with VO_2_ max.

**Characterization of left and right atrial function in healthy volunteers by cardiovascular magnetic resonance.**

Left and right atrial function changes with advancing age. In this study, Maciera et al. [59] measured maximal, preatrial contraction and minimal left and right atrial volumes in 120 healthy subjects (10 men/10 woman each decile). Gender had an impact on left and right atrial conduit and booster function while age was found to significantly impact most biatrial measures.

### Normative values – T1, ECV

**Comparison of different cardiovascular magnetic resonance sequences for native myocardial T1 mapping at 3T.**

A number of different CMR sequences have been advocated for native T1 mapping.. Comparison data among sequences is sparce at 3T. Teixeira and colleagues [60] compared shortened modified Look-Locker inversion recovery (ShMOLLI), modified Look-Locker inversion recovery (MOLLI) and single-shot acquisition (SASHA) sequences in phantoms and 40 healthy subjects with low or moderate cardiovascular risk at 3T. SASHA provided consistently greater native T1 in subjects and phantoms. On multivariate regression analysis, a longer T1 by MOLLI correlated with a lower left ventricular (LV) ejection fraction and female gender.

**Myocardial T1-mapping at 3T using saturation-recovery: reference values, precision and comparison with MOLLI.**

Myocardial T1 mapping has become an important quantitative tool for non-invasive tissue characterization for which minimal normative data at 3T are available. Weingartner et al. [61] compared balanced steady-state free precession saturation pulse prepared heart-rate independent inversion recovery (SAPPHIRE) and saturation-recovery single-shot acquisition (SASHA) T1 mapping with Modified Look-Locker Inversion recovery (MOLLI) in 20 healthy subjects (10 men). SAPPHIRE and SASHA yielded higher T1 time and lower extracellular volume fraction (ECV) compared with MOLLI. All three methods had similar interobserver variation for both T1 and ECV.

**Histologic validation of myocardial fibrosis measured by T1 mapping: a systemic review and meta-analysis.**

Myocardial fibrosis is being increasingly recognized as a common final pathway for a wide variety of cardiovascular diseases and validation histologic studies of T1 for fibrosis are limited. Diao and colleagues [62] performed a PubMed, EMBASE and Cochrane Library database search for studies applying T1 mapping to measure myocardial fibrosis that validated the results with histological data. A total of 15 studies including 308 patients who had both CMR and biopsy were identified. The pooled correlation between extracellular volume fraction (ECV) and T1 mapping was found to be 0.884 and was not notably heterogeneous.

#### Prognosis

The prognostic value of CMR [63–66] is being increasing recognized as adding value to patients with known or suspected cardiovascular disease.

**Left ventricular long axis function assessed during cine-cardiovascular magnetic resonance is an independent predictor of adverse cardiac events.**

Left ventricular long axis dysfunction is an early marker for many pathologic states. Rangarajan and colleagues [67] studied 400 consecutive patients undergoing CMR and measured mitral annular plane systolic excursion (MAPSE) in the 4-chamber view. During a median follow-up of 14.5 months, patients with a lateral MAPSE <1.11 experienced significantly higher adverse events; a remained a significant predictor after adjustment for established clinical risk factors (age, diabetes, hypertension, NYHA class, LV mass).

**Left ventricular long axis strain: a new prognosticator in non-ischemic dilated cardiomyopathy.**

Long axis strain represents global longitudinal left ventricular (LV) function. Riffel et al. [68] examined long axis strain in 146 patients with non-ischemic cardiomyopathy and found that long axis strain > − 5% showed a significant higher rate of adverse cardiac events (cardiac death, aborted sudden cardiac death, heart transplantation) independent of late gadolinium enhancement (LGE).

**Characterization and clinical significance of right ventricular mechanics in pulmonary hypertension evaluated with cardiovascular magnetic resonance feature tracking.**

Prognosis in pulmonary artery hypertension is related to right ventricular (RV) function. In this study, de Siqueira and co-workers [69] retrospectively identified 116 patients with pulmonary artery hypertension who underwent right heart catheterization within a month. Using feature tracking software, they found that RV peak global longitudinal strain was significantly reduced in the group with pulmonary hypertension and normal RV ejection fraction (vs. no pulmonary hypertension and normal RV ejection fraction). After adjustment for 6 clinically meaningful covariates, RV global longitudinal, global longitudinal strain rate, and global circumferential strain rate were independently associated with a composite end-point of all-cause mortality, lung transplantation or worsening NYHA functional class.

#### Coronary artery disease

CMR myocardial stress testing/perfusion [70–73] assessment as well as the use of CMR to assess myocardial infarction [74–78] continue to be examined in larger, multicenter studies.

### Myocardial infarction

**Cardiovascular magnetic resonance imaging of myocardial oedema following acute myocardial infarction: is whole heart coverage necessary?**

The area-at-risk is useful when assessing the efficacy of reperfusion therapy and novel cardioprotective agents after myocardial infarction. In this study by Hamshere and co-workers [79], CMR was performed in 167 patients after successful primary percutaneous coronary intervention with 82 patients undergoing a novel 3-short axis T2-STIR protocol and 85 both the novel 3-short axis protocol and a conventional 10 slice short-axis late gadolinium enhancement (LGE) protocol. The 3-slice T2-STIR and 10 slice LGE area-at-risk imaging showed a strong correlation with each other and with the angiographic risk scores.

**Long-term prognosis of unrecognized myocardial infarction detected with cardiovascular magnetic resonance in an elderly population.**

The long-term prognosis of CMR detected unrecognized myocardial infarction is not fully evaluated. Barbier et al. [80] performed late gadolinium enhancement (LGE) CMR in 248 randomly chosen 70 year-olds. During a mean 11 year follow-up, adverse cardiac events occurred in 10% of individuals without infarction scar on CMR, 20% of subjects with unrecognized CMR infarction, and 45% of individuals with known infarction.

**Scar quantification by cardiovascular magnetic resonance as an independent predictor of long-term survival in patients with ischemic heart failure treated by coronary artery bypass graft surgery.**

Late gadolinium enhancement (LGE) scar burden is associated with inversely associated with functional recovery after coronary artery bypass graft surgery (CABG). In this study, Kancharla et al. [81] identified 196 patients who had undergone CMR prior to CABG, of whom scar was present in 72%. Over a median follow-up of 8.3 years, there were 64 deaths. There was no significant difference in mortality in the LGE(+) and LGE(−) group (29% vs. 37%, respectively) but in the group with scar, a lower scar burden was independently associated with increased survival.

**Antecedent hypertension and myocardial injury in patients with reperfused ST-elevation myocardial infarction.**

Antecedent hypertension is associated with poor outcome in patients with ST elevation myocardial infarction (STEMI). Reinstadler et al. [82] studied 792 consecutive STEMI patients reperfused within 12 h of symptom onset. Antecedent hypertension was present in 540 (68%) of patients and was associated with major adverse cardiac events (MACE). There were, however, no significant difference in the area at risk, infarct size, myocardial salvage index, extent of microvascular obstruction, and left ventricular ejection fraction (LVEF) between groups.

**Infarct size following complete revascularization in patients presenting with STEMI: a comparison of immediate and staged in-hospital non-infarct related artery PCI subgroups in the CvLPRIT Study.**

The CvLPRIT Study showed a trend for improved clinical outcomes in the complete revascularization group in those treated with immediate vs. staged in-hospital approach for multivessel coronary artery disease (CAD) and acute STEMI. Khan and co-workers [83] report on the 93 patients in the CMR substudy of CvLPRIT (63 immediate/30 staged). Patients treated with the staged approach had more visible left ventricular (LV) thrombus and greater incidence of no-reflow. After adjustment for confounders, staged patients had larger infarct size and lower LV ejection fraction (LVEF).

### Stress CMR

**Perfusion cardiovascular magnetic resonance and fractional flow reserve in patients with angiographic multi-vessel coronary artery disease.**

Non-invasive CMR perfusion CMR and invasive fractional flow reserve (FFR) are emerging as the most accurate tools for assessment of myocardial ischemia. In this study, Hussain and co-workers [2] performed 1.5T CMR and FFR in 41 patients with angiographic 2 or 3-vessel coronary artery disease. On a per-patient basis, CMR and FFR detected identical ischemic territories in 19 (46%) patients. On a per-vessel basis, there was concordance in 72% of 123 territories, with CMR identifying fewer or greater ischemic territories in 34% and 12%, respectively.

**Quantitative assessment of myocardial blood flow in coronary artery disease by cardiovascular magnetic resonance: comparison of Fermi and distributed parameter modeling against invasive methods.**

Absolute quantification of myocardial blood flow (MBF) may improve the diagnosis and prognostication of obstructive coronary artery disease (CAD). In this pilot study, Papanastasiou et al. [84] studied 28 subjects with known or suspected CAD with adenosine stress 3 T CMR. Data were analyzed using the Fermi and distributed parameter modeling. On receiver operator curve (ROC) analysis, the distributed parameter model outperformed the conventional Fermi model on a per vessel and per patient.

**Benefits of chronic total coronary occlusion percutaneous intervention in patients with heart failure and reduced ejection fraction: insights from a cardiovascular magnetic resonance study.**

Chronic total occlusion percutaneous coronary intervention can improve angina and left ventricular (LV) ejection fraction (LVEF). Cardona and co-workers [85] studied 29 patients with heart failure and reduced ejection fraction and evidence of viability and/or ischemia in a territory supplied by a chronic total occlusion who were successfully treated with a percutaneous coronary intervention. CMR was performed prior to and 6 months after intervention. At 6 months, there was a decrease in LV end-systolic volume and an increase in LVEF. In addition, the number of LV segments demonstrating ongoing perfusion was diminished. Angina and NYHA functional class improved and BNP levels declined.

## Technical developments

CMR technical advances continue to dominate the field and the *JCMR* publications. Advances are both in sequence development [86–89], T1 mapping and extracellular volume fraction (ECV) [90–98], T2/T2* mapping [98–104], arrhythmia/motion correction [105–110], 3T [111, 112], flow quantification [113–120], perfusion [121, 122], deformation [123–132], strain [133], diffusion tensor imaging [134] and phantoms [135].

### Automated analysis

**Improved workflow for quantification of left ventricular volumes and mass using free-breathing motion corrected cine imaging.**

Traditional breath-hold cine CMR can be problematic. Free breathing alternatives have relied on multiple averages or real-time imaging. The use of distributed computing was recently proposed as a way to improve clinical workflow with such algorithms. Cross et al. [136] studied 25 patients and 25 healthy subjects with free breathing with averaging and breath-hold balanced steady state free precession (bSSFP) compared with motion corrected re-binning. Motion corrected re-binning and averaged free-breathing compared favorably with bSSFP for left ventricular (LV) mass, end-diastolic volume (EDV) and end-systolic volume (ESV). Both motion corrected re-binning and averaged free-breathing SSFP acquisitions and reconstruction times were shorter than the breath-hold bSSFP method – with an average 37%/3 min shorter time for motion corrected re-binning (vs breath-hold SSFP).

**Systolic MOLLI T1 mapping with heart-rate dependent pulse sequence sampling scheme is feasible in patients with atrial fibrillation.**

The irregular rhythm of atrial fibrillation (AF) may cause inaccurate T1 estimation due to mis-triggering and inadequate magnetization recovery. Zhao et al. [137] used systolic T1 mapping with a heart-rate dependent pulse sequence to overcome this issue. Thirty patients with AF and 13 healthy subjects underwent 3T CMR using a modified Look-Locker Inversion Recovery (MOLLI) sequence. For AF patients, both the fixed and the heart rate dependent sampling scheme were performed in systole and diastole. In healthy subjects, the native T1 and ECV generated from the fixed sampling scheme were lower than the heart-rate dependent and 2nd fixed sampling scheme. In AF patients, more T1 mapping artifacts were found in diastole than in systole. The overall left ventricular (LV) T1 time and ECV were greater with diastolic acquisitions.

**Evaluation of an automated method for arterial input function detection for first pass myocardial perfusion cardiovascular magnetic resonance.**

Quantitative assessment of myocardial blood flow (MBF) with first-pass perfusion CMR requires a measurement of the arterial input function. In this study, Jacobs et al. [138] propose an automated method to improve the objectivity and reduce processing time. First-pass rest and stress perfusion CMR data were analyzed from 270 clinical studies. Automated imaging processing steps included motion correction, intensity correction, detection of the left ventricle (LV), independent component analysis, and LV pixel thresholding to calculate the arterial input function. Data were compared with manual reference measurements. Their proposed method was successfully processed in 99.63% of the images. Manual and automated arterial input function were highly correlated and required less processing time that the manual approach with similar myocardial blood flow estimates.

Validation of T2* in-line analysis for tissue iron quantification at 1.5T.

There is a need for a simple on-line T2* analysis for T2* so as to improve analysis reproducibility, especially with low volume centers. In this study, Alam and co-workers [139] compared a clinically validated T2* method and a novel works-in-progress sequence with in-line T2* measurements in 78 iron overload patients and 22 healthy subjects. Liver T2* varied from 0.8 to 35.7 ms and cardiac T2* from 6.0 to 52.3 ms. The novel in-line method had difficulty with accurate delineation of the septum due to artifacts and had some overestimation due to the inability to manually correct for noise by truncation of erroneous later echo times. Reproducibility for the existing method was superior to the in-line method.

**The effects of extracellular contrast agent (gadobutrol) on the precision and reproducibility of cardiovascular magnetic resonance feature tracking.**

CMR feature tracking is an area receiving considerable interest. In this study, Kuetting and colleagues [140] perform cine mid-ventricular short axis and horizontal long axis cine 1.5T CMR in 40 healthy subjects before and 10-15 min after injection of a double dose of gadobutrol. Feature tracking derived basal, mid and apical peak systolic circumferential strain, peak longitudinal strain, and midventricular epicardial and mid-ventricular peak systolic circumferential strain rate were all *reduced* after gadobutrol . Post-contrast strain assessment also showed higher intra and inter-observer variability.

**Accelerated two-dimensional cine DENSE cardiovascular magnetic resonance using compressed sensing and parallel imaging.**

Cine displacement encoding and stimulated echoes (DENSE) provides accurate quantitative imaging of cardiac mechanics with rapid displacement and strain analysis, but image acquisition times are relatively long. In this study, Chen et al. [141] describe an accelerated cine DENSE sequence with variable-density spiral k-space sampling and golden angle rotations through time. A compressed sensing method, Block Low-rank Sparsity with Motion-guidance (BLOSM) was also combined with sensitivity encoding (SENSE). For retrospectively-under sampled data, BLOSM-SENSE provided similar or lower root mean square error at rates 2 at rate-2 and lower at rate-4 acceleration compared with SENSE.

Comparison of 3T and 1.5T for T2* magnetic resonance of tissue iron.

T2* CMR tissue iron concentration has improved the outcome of transfusion dependent anemia patients. In this study, Alam and co-workers [142] performed 1.5T and 3T T2* CMR in 104 transfusion dependent patients and 20 healthy subjects. Association between heart and liver T2* at 1.5T and 3T were non-linear and R2* approximately doubled at 3T with linear fits for both heart and liver. Coefficients of variation for intra and inter-observer reproducibility as well as inter-study reproducibility tended to be better at 1.5T. Artifact scores were also significantly worse at 3T black blood.

**The impact of hematocrit on oxygenation-sensitive cardiovascular magnetic resonance.**

Oxygen sensitive CMR is a promising technique in which images are generated through tissue deoxyhemoglobin which is negatively correlated with signal intensity. In this study, Guensch et al. [143] performed oxygen sensitive CMR in 21 healthy subjects using vasoactive breathing stimuli and repeated after rapid infusion of 1 L of lactated Ringer’s solution. Rapid infusion resulted in a fall in hemoglobin while baseline myocardial signal intensity increased, and in males, there was a strong association between the change in hemoglobin concentration and percent change in signal intensity.

**Ferumoxytol-enhanced magnetic resonance imaging methodology and normal values at 1.5T and 3T.**

Ultrasmall supraparamagnetic particles of iron oxide (USPIO)-enhanced MRI can detect tissue-resident macrophage activity and thereby identify focal cellular inflammation. Stirrat and co-workers [144] studied 20 healthy subjects who underwent late gadolinium enhancement (LGE) imaging at baseline and t2* imaging 24 h after USPIO infusion. Following USPIO, there were changes in R2* at 1.5T in the myocardium, skeletal muscle, kidney, liver, spleen and blood; and at 3T in the myocardium, kidney liver, spleen blood and bone. Tissues showing the greatest ferumoxytol enhancement were the reticuloendothelial system: liver, spleen, and bone marrow.

**Robust free-breathing SASHA T1 mapping with high-contrast image registration.**

Many widely used myocardial native T1 mapping sequences use breath-hold acquisitions that limit the precision of calculated T1 maps. In this study, Chow et al. [145] propose a novel method for generating high-contrast SAturation-recovery single-SHot Acquisition (SASHA) images to enable a robust image registration approach to free breathing T1 mapping. Breath-hold SASHA, high-contrast SASHA and MOdified Look-Locker Inversion recovery (MOLLI) images were acquired in 10 subjects. Myocardial T1 from free breathing high-contrast SASHA were similar to breath-hold SASHA. In addition, T1 map quality scores were superior with free breathing high-contrast SASHA.

**Compressed sensing real-time cine cardiovascular magnetic resonance: accurate assessment of left ventricular function in a single breath-hold.**

Cine CMR accelerated by compressed sensing is used to assess left ventricular (LV) function. Kido and co-workers [146] performed conventional breath-hold cine and breath-hold real-time compressed sensing cine CMR study to obtain a short-axis stack of 8 contiguous images. Total imaging time was shorter with compressed sensing, though compressed sensing had reduced image quality. Conventional and compressed sensing had similar quantitative metrics for all measurements (end-diastolic volume, end-systolic volume, stroke volume, ejection fraction, and mass).

**An interactive videogame designed to improve respiratory navigator efficiency in children undergoing cardiovascular magnetic resonance.**

Many CMR sequences have long scan durations that necessitate respiratory navigator gating, but breathing patterns are more often inconsistent in children. To address this, Hamlet and colleagues [147] developed a custom software that processed the respiratory navigator image in real-time and provided diaphragmatic position to the patient as a cartoon avatar as visual feedback. Using this approach, average navigator efficiency improved from 33% to 58% and signal-to-noise ratio improved by 5%. There was no difference in either metric between trained and untrained participants.

**Flow measurement at the aortic root – impact of location of through-plane phase contrast velocity mapping.**

CMR aortic flow is often measured at the sinotubular junction even though placement of the slice just above the aortic valve may be more precise. Bertelsen et al. [148] studied 121 patients >70 years by placing the slice at the sinotubular and valve levels. Overall, stroke volume measured at the sinotubular junction was 13-16% *lower*. Among the 58 patients without any valvulopathy, stroke volume measured at the valve level was closest to that measured by left ventricular stroke volume, but still significantly lower than volumetric values.

**A medical device-grade T1 and ECV phantom for global T1 mapping quality assurance – the T1 mapping and ECV Standardization in cardiovascular magnetic resonance (T1MES) program.**

T1 mapping and extracellular volume (ECV) have the potential to diagnose disease and monitory therapies, but measurements differ between scanners and pulse sequences. Captur and co-workers [149] designed a phantom incorporating nine clinically relevant ranges of T1 and T2 in blood and myocardium, pre and post-contrast and 1.5 T and 3 T. The coefficient of variation was 1% or less between repeat scans indicating good short-term reproducibility. Reproducible manufacture was established and the device received regulatory clearance from the United States Food and Drug Administration (FDA) and the Conformite Europeene (CE) marketing.

**Hemodynamic evaluation in patients with transposition of the great arteries after the arterial switch operation: 4D flow and 2D phase contrast cardiovascular magnetic resonance compared with Doppler echocardiography.**

Peak velocity measurements are used to evaluate stenosis in patients with transposition of the great arteries after the arterial switch operation. Jarvis and co-workers [150] performed 4D flow and 2D phase contrast CMR in 19 patients 12-25 years after arterial switch operation for transposition. Data were compared with Doppler echocardiography. Significantly higher peak velocities were found with 4D flow vs 2D phase contrast, with no significant difference between 4D flow and Doppler measurements.

**D’Errico L, Lamacie MM, Juan LJ, et al. Effects of slice orientation on reproducibility of sequential assessment of right ventricular volumes and ejection fraction: short-axis vs. transverse SSFP cine cardiovascular magnetic resonance.**

Reproducibility of right ventricular (RV) volumes and function are of utmost importance, but the optimal slice orientation for RV measurements is unknown. In this study, D’Errico et al. [151] performed cine CMR in the ventricular short axis and transverse slice orientations in addition to phase velocity mapping of the main pulmonary artery in 18 subjects. Both short axis and transverse imaging slices were found to provide similarly reliable and reproducible measures and thus suitable for baseline and follow-up studies.

**Magnetic resonance imaging phantoms for quality-control of myocardial T1 and ECV mapping: specific formulation, long-term stability and variation with heart rate and temperature.**

CMR phantoms are needed for quality assurance, but their long-term stability for verification of myocardial T1 and extracellular volume fraction (ECV) are unknown. In this study, Vassilous et al. [152] examined nickel-chloride agarose gel phantoms to mimic blood and myocardial T1, T2, and post-gadolinium T1 and ECV. They found only small relative changes in all the native and post-gadolinium T1 values (up to 9.0%) and ECV (up to 8.3%) over a 12 month period. Native and post-gadolinium T2 had a < 2% change. Temperature sensitivity showed the MOLLI T1 values in the long T1 phantoms increasing by 23.9 ms per degree increase and short T1 phantoms increasing by 0.3 ms per degree increase. There was also a very small increase in ECV with temperature increase.

**A clinical combined gadobutrol bolus and slow infusion protocol enabling angiography, inversion recovery whole heart, and late gadolinium enhancement imaging in a single study.**

The ability to perform 3D inversion recovery CMR angiography and late gadolinium enhancement (LGE) in the same sequence is desirable. Tandon and co-workers [153] propose the use of a bolus of 0.1 mmol/kg gadobutrol for the time resolved CMR angiogram followed by a slow infusion of 0.02-0.03 ml/s with image navigated 3D inversion recovery balanced steady state free precession initiated 45-60 s after the infusion onset. Data from 10 consecutive pediatric subjects were retrospectively assessed and found to have good image quality.

**Improved dark blood imaging of the heart using radial balanced steady state free precession,**

Dark blood CMR imaging is typically performed using a breath-hold, dual inversion Cartesian fast spin-echo pulse sequence. In this study, Edelman et al. [154] implemented a novel radial balanced steady state free precession (bSSFP) pulse sequence and examined 6 healthy subjects and 27 patients referred for CMR. In both groups, the single shot dual inversion radial bSSFP images showed fewer motion artifacts with faster acquisition.

**Dark blood late gadolinium enhancement.**

Bright blood late gadolinium enhancement (LGE) displays excellent contrast between infarcted and normal myocardium, but the contrast between the infarcted myocardium and blood pool is often suboptimal. To address this, Kellman and colleagues [155] developed a black blood LGE sequence in which an inversion recovery T2 preparation pulse was combined with a single shot steady state free precession imaging and respiratory corrected averaging to achieved dark blood LGE images. Thirty patients with subendocardial infarction were studied with the bright blood and dark blood LGE methods. The contrast-to-noise ratio (CNR) of the dark blood LGE method was 13% lower than the bright blood method, but the CNR between the infarction and blood pool was positive for all of the dark blood cases and was negative for 63% of the bright blood cases.

**Feasibility of cardiovascular magnetic resonance derived coronary wave intensity analysis.**

Wave intensity analysis of the coronary arteries allows for description of the predominant mechanisms influencing coronary flow during the cardiac cycle. Raphael et al. [156] performed wave intensity analysis by CMR and compared data with invasive Doppler data in 12 arteries (8 left; 4 right). The combination of CMR-derived pressure and velocity data produced the expected pattern of forward and reverse compression and expansion waves with good correlation with invasive data and good intra-study CMR reproducibility.

### Diffusion tensor imaging

**Relationship between cardiac diffusion tensor imaging parameters and anthropometrics in healthy volunteers.**

In-vivo cardiac diffusion tensor imaging (cDTI) is uniquely capable of non-invasively interrogating laminar myocardial dynamics. In this study by McGill and co-workers [157], 3 T cDTI was performed in 43 subjects during the systolic and diastolic pauses with assessment of global and regional fractional anisotropy, mean diffusivity (MD), helix angle gradient (HAg) and the secondary eigenvector angulation (E2A). All cDTI parameters displayed regional heterogeneity and the heart rate had a significant but clinically small impact on systolic values. Male sex and increasing left ventricular (LV) end-diastolic volume were associated with increased systolic HAg, while diastolic HAg and systolic E2A were both directly related to LV mass and body surface area.

**In vivo cardiovascular magnetic resonance of 2D vessel wall diffusion anisotropy in carotid arteries.**

Diffusion weighted CMR has shown great potential in discriminating between healthy and diseased vessel tissue by evaluating the apparent diffusion coefficient (ADC). Opriessnig et al. [158] performed high resolution CMR diffusion tensor imaging (DTI) in 12 healthy male subjects at 3 T and found the tangential component as the principle direction of diffusion. Mean vessel wall fractional anisotropy values ranged from 0.7 for the youngest to 0.56 for the oldest subject with a significant linear relationship between fractional anisotropy and age. In addition, a linear trend was seen between radial diffusivity and age.

## Vascular imaging

Extension of CMR beyond the heart is increasingly being recognized as important in the complete evaluation of patients with cardiac disease. These include vessel wall/plaque imaging [159, 160] as well as vascular stiffness/compliance [161–167], angiography [168], and limb perfusion [169].

**Expansive arterial remodeling of the carotid arteries and its effect on atherosclerotic plaque composition and vulnerability: an in-vivo black-blood 3T CMR study in symptomatic stroke patients.**

Expansive remodeling is a feature of vulnerable atherosclerotic plaque. In this study by Saam and co-workers [170], 111 symptomatic patients with acute unilateral ischemic stroke and carotid plaques of at least 2 mm thickness underwent multisequence black-blood 3T CMR of the proximal internal carotid arteries. 78% of the 202 arteries examined showed evidence for atherosclerotic disease with American Heart Association (AHA) lesion type III or higher. They also found significant correlations of the modified remodeling index with lumen area, wall area, vessel area, and wall thickness.

**Nonenhanced hybridized arterial spin labeled magnetic resonance angiography of the extracranial carotid arteries using a fast low angle shot readout at 3 Tesla.**

Disorders of the extracranial carotid arteries are frequently evaluated by contrast-enhanced CMR angiography, but gadolinium contrast is contraindicated in patients with moderate to severe renal insufficiency due to concerns of nephrogenic systemic fibrosis. In this retrospective report, Kokzoglou and co-workers [171] examine image quality of non-enhanced hybridized arterial spin labeling and contrast enhanced CMR angiography in 37 patients presenting with neurologic symptoms. Contrast enhanced CMR angiography provided the best image quality, while the non-enhanced hybridized arterial spin labeling approach provided image quality that exceeded 2D time-of-flight imaging at the carotid bifurcation and internal and external carotid arteries. Further, all 9 vascular abnormalities of the carotid and intracranial arteries were detected by contrast enhanced CMR angiography and hybrid arterial spin labeling, with no false positives.

**Assessment of aortic stiffness by cardiovascular magnetic resonance following the treatment of severe aortic stenosis by TAVI and surgical AVR.**

Aortic stiffness is increasingly recognized as an independent predictor of adverse cardiovascular outcomes. In this study, Al Musa and co-workers [172] performed 1.5 T CMR in 72 patients before and 6 months after surgical aortic valve replacement and transcutaneous aortic valve implantation (TAVI). At 6 months, surgical aortic valve replacement was associated with a significant *decrease* in ascending aortic distensibility/*increased* ascending aortic stiffness and an *increase* in pulse wave velocity. In contrast, no significant change was noted in the (older) TAVI group.

**Semi-automatic carotid intraplaque hemorrhage detection and quantification on Magnetization-Prepared Rapid Acquisition Gradient-Echo (MP-RAGE) with optimized threshold selection.**

Intraplaque hemorrhage is associated with progression of atherosclerosis and cardiovascular events. Liu and colleagues [173] studied 14 patients scheduled for carotid endarterectomy using MP-RAGE CMR. The presence and area of intraplaque hemorrhage was determined by histology. Optimized intensity thresholds for intraplaque hemorrhage were 1.0× the sternocleidomastoid muscle intensity, 1.6× the adjacent muscle intensity, and 2.2 times the median intensity. Using a semi-automated method with these thresholds, the intraplaque hemorrhage detection sensitivity was 59% and specificity 85%.

**Aortic stiffness and its impact on left atrial volumes and function in patients after successful coarctation repair: a multiparametric cardiovascular magnetic resonance study.**

As compared with a generally good prognosis for early repair, increased cardiovascular morbidity is present in adults with late aortic coarctation repair. To examine potential causes, Voges et al. [174] performed CMR in 51 patients (median 17 years) 14 +/− 7.5 years after coarctation repair and identified reduced thoracic aortic repair in the entire group. In addition, descending aorta distensibility and pulse wave velocity correlated negatively with age at the time of coarctation repair.

**The diagnostic value of non-contrast enhanced quiescent interval single shot (QISS) magnetic resonance angiography at 3T for lower extremity peripheral arterial disease, in comparison to CT angiography.**

The high incidence of renal insufficiency in patients with peripheral arterial disease raises the need for non-contrast peripheral arterial options. In this study, Wu and colleagues [175] examined the diagnostic performance of quiescent interval single shot (QISS) CMR angiography at 3 T as compared with computed tomography (CT) angiography in 32 consecutive patients with suspected peripheral arterial disease. QISS CMR angiography was slightly inferior to CT angiography for image quality with similar overall sensitivity and specificity for disease. However, in heavily calcified segments, QISS CMR angiography was superior to CT angiography.

**Improved high-resolution pediatric vascular cardiovascular magnetic resonance with gadofosveset-enhanced 3D respiratory navigated, inversion recovery prepared gradient echo readout imaging compared to 3D balanced steady-state free precession readout imaging.**

Improved definition of vascular structures is a common indication for CMR in the pediatric population with non-contrast 3D respiratory navigated, T2-prepared fat saturation imaging with a balanced steady state free precession (bSSFP) readout is commonly used. Tandon et al. [176] propose an alternative 3D gradient echo inversion recovery sequence with a blood pool agent, gadofosveset trisodium and examined 35 patients with both sequences. The contrast-enhanced 3D inversion recovery sequence had superior overall image quality (including patients with intrathoracic metal) for the right coronary artery, pulmonary arteries/veins and blood pool homogeneity. Signal-to-noise (SNR) and contrast-to-noise (CNR) were also superior for the left atrium and left ventricle, but not the pulmonary arteries.

**Co-existing intracranial and extracranial carotid artery atherosclerosis plaques and recurrent stroke risk: a three-dimensional multicontrast cardiovascular magnetic resonance study.**

As a systemic disease, atherosclerosis commonly affects intracranial and extracranial carotid arteries simultaneously. In this study, Xu and colleagues [177] examined 58 patients with recent anterior circulation cerebrovascular symptoms and at least one carotid plaque for multiparametric CMR. Co-existing intracranial and extracranial plaque was found in 78%. For those with stroke, the presence of both intracranial and extracranial plaque was associated with recurrent stroke, even after adjusting for traditional clinical risk factors.

## Animal models

Small and large animal models continue to play an important role in advancing our understanding of cardiovascular disease [178–185].

**Myocardial T1 maps reflect histological findings in acute and chronic stages of myocarditis in a rat model.**

CMR offers both diagnostic and prognostic information for myocarditis. In this rat model study, Jeuthe and co-workers [186] immunized male young Lewis rats with porcine myocardial myosin and performed native and contrast-enhanced CMR prior to and on days 14, 21, and 35. All immunized rats developed myocarditis with histologic wall thickening and biventricular macrophage-rich mixed inflammatory infiltrates. CMR demonstrated increased native myocardial T1 and decreased post-contrast T1.

**A new automatic algorithm for quantification of myocardial infarction imaged by late gadolinium enhancement cardiovascular magnetic resonance: experimental validation and comparison to expert delineations in multi-center, multi-vendor patient data.**

Late gadolinium enhancement (LGE) CMR using inversion recovery or phase sensitive inversion recovery (PSIR) has become one of the most widely employed CMR clinical sequences in the assessment of patients with known or suspected coronary artery disease. However, there is no clinical standard for infarction quantification. In this study, Engblom and colleagues [187] apply a novel automated algorithm on data derived from 7 swine and with 124 patients from a multi-center, multi-vendor ST elevation study. Infarct size by the automated algorithm in swine showed a slight bias to ex-vivo triphenyltetrazolium chloride (TTC) staining while the patient study showed the automated algorithm showed a slight bias to expert delineation.

**Cardiovascular magnetic resonance detectgs the progression of impaired myocardial perfusion reserve and increased left-ventricular mass in mice fed a high-fat diet.**

Impaired myocardial perfusion reserve (MPR) is prevalent in obesity and diabetes even in the absence of obstructive coronary artery disease. Naresh and co-workers [188] studied C57Bl/6 mice fed a high-fat or low-fat diet and imaging at 6, 12, 18, and 24 weeks post-diet. Body weight, left ventricular (LV) mass and wall thickness were increased and MPR reduced in the high-fat cohort at 18 and 24 weeks. Coronary artery vascular reactivity to adenosine and acetylcholine was also reduced in the high-fat cohort.

**Early-stage heart failure with preserved ejection fraction in the pig: a cardiovascular magnetic resonance study.**

A hypertensive deoxy-corticosterone acetate salt-treated pig model of early stage heart failure with preserved ejection fraction (HFpEF). Reiter and colleagues [189] studied 5 HFpEF swine and 6 controls with dobutamine stress at 3 T. They found the HFpEF swine had increased left ventricular (LV) mass and wall thickness along with increase left atrial volume. Myocardial perfusion reserve was decreased in the HFpEF swine. T1 time did not differ between groups.

## Congenital heart disease

The non-ionizing multiparametric attributes of CMR make it ideal for imaging of the pediatric and adult patient with known or suspected congenital heart disease [190–194] including blood flow [195]).

### Tetralogy of Fallot

**Vicious circle between progressive right ventricular dilation and pulmonary regurgitation in patients after tetralogy of Fallow Repair. Right heart enlargement promotes flow reversal in the left pulmonary artery.**

The left pulmonary artery contributes more than the right pulmonary artery to total pulmonary regurgitation in patients after tetralogy of Fallot (TOF) repair. Kato and co-workers [196] studied 48 patients with TOF repair and found no difference in left and right pulmonary artery diameters, but the left pulmonary artery had less total forward flow, smaller net forward flow, and greater regurgitant fraction. There was no difference in regurgitant flow volume. Indexed right ventricular end-diastolic volume correlate4d with left pulmonary artery regurgitant fraction, but not with right pulmonary artery regurgitant fraction.

**Left and right ventricular dyssynchrony and strains from cardiovascular magnetic resonance feature tracking do not predict deterioration of ventricular function in patients with repaired tetralogy of Fallot.**

Patients with repaired tetralogy of Fallot (TOF) suffer from progressive ventricular dysfunction decades after surgical repair. In this study, Jing et al. [197] retrospectively identified 153 repaired TOF patients who had at least 2 CMRs performed >6 month apart without intervening mechanical intervention. After a mean follow-up of 2.9 years, no feature tracking metric (interventricular dyssnchrony, left ventricular (LV) and right ventricular (RV) peak global circumferential strain and LV and RV peak global longitudinal strain) was associated with adverse outcomes or ventricular remodeling.

**Increased left ventricular myocardial extracellular volume is associated with longer cardiopulmonary bypass times, biventricular enlargement and reduced exercise tolerance in children after repair of Tetralogy of Fallot.**

Unfavorable left ventricular (LV) remodeling may be associated with adverse remodeling after repair of tetralogy of Fallot (TOF). In this cross-sectional, prospective study, Riesenkampff and co-workers [198] studied 31 TOF repair subjects and 15 controls and found no difference in native T1 or extracellular volume fraction (ECV) between TOF and controls, though the female TOF cohort had a higher ECV than male TOF cohort. In the TOF group, higher native T1 and ECV correlated with higher Z-scores of right ventricular (RV) and LV end-diastolic value but not with reduced LV or RV ejection fraction. Higher native T1 did correlate with worse LV longitudinal and mid short axis circumferential strain.

## Miscellaneous topics/reviews

CMR assessment of blood flow [199] and image processing [200] are likely to play an increasing role in the assessment of our patients and automated analyses of the growing image datasets. Helping clinicians understand the role of CMR amongst all of the cardiovascular imaging will also be of increasing importance.

**Physician’s professional identities: a roadmap to understanding “value” in cardiovascular imaging.**

Quality improvement efforts have been challenged by limited adoption of initiatives and policies. In this study, Keller and co-workers [201] interviewed 15 Northwestern Medicine physicians from internal medicine, cardiology, emergency medicine, cardiac/vascular surgery, and radiology disciplines. Differences in perceived cardiovascular imaging value and guideline use were explained by three value associated categories (managers; diagnosticians; and fixers) that were further differentiated along three axes (broad v. focused-thinkers; complex v. definitive-answer seekers; and public visibility).

**Use of self-gated radial cardiovascular magnetic resonance to detect and classify arrhythmias (atrial fibrillation and premature ventricular contraction).**

Arrhythmias can degrade CMR image quality. As a result, automated detection and sorting of the most frequent types of arrhythmias may improve image quality. Piekarski and colleagues [202] retrospectively studied 111 patients who underwent self-gated free-breathing radial cardiac cine CMR with compressed sensing reconstruction for detection of atrial fibrillation, premature ventricular contractions, and non-sinus rhythm to detect non-sinus rhythm. Using their algorithm to identify non-sinus rhythm, sensitivity, specificity and accuracy was 93%, 95% and 94% respectively to discriminate non-arrhythmic and arrhythmic patients and 83%, 71% and 77% to discriminate atrial fibrillation and premature ventricular contractions.

**Top 100 cited articles in cardiovascular magnetic resonance: a bibliometric analysis.**

Bibliometric studies can help guide researchers and research funding agencies toward areas where allocation or increase in research activity is warranted. Khan and co-workers [203] performed a Web of Science search to identify all CMR publications to identify the top 100 cited articles. The vast majority (86%) of manuscripts were published between 2000 and 2014 and 17 articles were cited more than 500 times. A total of 52% were from the United States and 21% from the United Kingdom. *Circulation* and *the Journal of the American College of Cardiology* published 62% of the articles.

**Myocardial triglyceride content at 3T cardiovascular magnetic resonance and left ventricular systolic function: a cross-sectional study in patients hospitalized with acute heart failure.**

Increased myocardial triglyceride (TG) content has been recognized as a risk factor for cardiovascular disease. Liao et al. [204] performed 3 T CMR 1H spectroscopy in 50 patients (including 25 with left ventricular ejection fraction <50%) discharged after hospitalization for acute heart failure and 21 age and sex matched controls. Myocardial unsaturated fatty acid/water ratio was found to be differ (0.79% vs. 0.21% vs. 0.14%) between the low ejection fraction heart failure, normal ejection fraction heart failure and healthy controls, respectively.

## Reviews

Focused reviews are an important component of the *JCMR* educational process, especially with the rapid advances made in the CMR field. In 2016, reviews were published on myocardial spin labeling [205], extracardiac findings seen on CMR [206], feature tracking [207], Takotsubo cardiomyopathy [208], T1 and ECV [209].

## Case report

Detection of metallic cobalt and chromium liver deposition followed failed hip replacement using T2* and R2 magnetic resonance.

In 2016, the *JCMR* had a single case report of a patient with cobalt and chromium liver deposition following a failed hip replacement [210]. It is not anticipated that there will be any case reports in 2017 and beyond. Those interested in publishing a CMR case report are encouraged to submit their work to the case series on the SCMR web site (www.scmr.org).

## Abbreviations

ADC: Apparent diffusion coefficient
AF: Atrial fibrillation
APC: Author processing charge
ARVC: Arrhthomogenic right ventricular cardiomyopathy
BNP: Brain natriuretic peptide
BSA: Body surface area
bSSFP: balanced steady state free precession
CAD: Coronary artery disease
CNR: Contrast-to-noise ratio
CVD: Cardiovascular disease
DENSE: Displacement encoding and stimulated echoes
DTI: Diffusion tensor imaging
ECV: Extracellular volume fraction
EDV: End-diastolic volume
ESV: End-systolic volume
FFR: Fractional flow reserve
HCM: Hypertrophic cardiomyopathy
HFpEF: Heart failure with preserved ejection fraction
JCMR: Journal of Cardiovascular Magnetic Resonance
LGE: Late gadolinium enhancement
LV: Left ventricle/left ventricular
LVEF: Left ventricular ejection fraction
LVNC: Left ventricular non-compaction
MACE: Major adverse cardiac event
MAPSE: Mitral annular plane systolic excursion
MBF: Myocardial blood flow
MOLLI: Modified Look-Locker inversion recovery
MPR: Myocardial perfusion reserve
NYHA: New York Heart Association
QISS: Quiescent interval single shot
RV: Right ventricle/right ventricular
SASHA: Single shot acquisition
SCMR: Society for Cardiovascular Magnetic Resonance
SENSE: Sensitivity encoding
ShMOLLI: Shortened modified Look-Locker inversion recover
SNR: Signal-to-noise ratio
STEMI: ST elevation myocardial infarction
TAVI: Transcutaneous aortic valve implantation
TOF: Tetralogy of Fallot
USPIO: Ultrasmall particles of iron oxide
